# Extracellular mass to body cell mass ratio association to nutritional and functional status in middle-aged and older adults

**DOI:** 10.3389/fnut.2025.1683856

**Published:** 2025-11-18

**Authors:** Antonella Cano, Lucia Ventura, Maria Grazia Catte, Elena Castiglia, Josetta Sanna, Gianluca Martinez, Carmen Oneto, Nicola Loi, Francesca Ginatempo, Andrea Manca, Franca Deriu

**Affiliations:** 1 Azienda Ospedaliero Universitaria di Sassari, AOUSS, Sassari, Italy; 2 Department of Biomedical Sciences, University of Sassari, Sassari, Italy; 3 Unit of Endocrinology, Nutritional and Metabolic Disorders, Azienda Ospedaliero Universitaria di Sassari, AOUSS, Sassari, Italy

**Keywords:** extracellular mass to body cell mass ratio, body composition, mini nutritional assessment, aging, muscle quality index, prognostic nutritional index, geriatric nutritional risk index

## Abstract

**Introduction:**

Poor nutrition and progressive loss of muscle mass is frequent in older individuals. Extracellular mass to body cell mass ratio (ECM/BCM), an easy to calculate index obtained by bioelectrical impedance vector analysis (BIVA), is a parameter of catabolism or extracellular mass expansion, mainly studied in pathological conditions. The aim of the study was to characterize ECM/BCM ratio and its association to indexes of nutritional and functional status in middle-aged and community dwelling older individuals.

**Methods:**

Participants aged 51–89 years old (*n* = 158, 96 women) were included in the study. Comorbidity burden was assessed by Charlson comorbidity index. Nutritional status was evaluated by mini nutritional assessment (MNA) and BIVA. Plasma biochemical parameters were measured, and prognostic nutritional index (PNI) and geriatric nutritional risk index (GNRI) were calculated. Functional performance was assessed by hand dynamometry and evaluated by means of muscle quality index (MQI).

**Results:**

ECM/BCM mean value was 0.91 ± 0.12. Higher values were observed in women and in individuals over 75. Subjects displaying ECM/BCM above median, presented significantly lower MNA score, PNI, GNRI, and MQI (all *p* ≤ 0.05), compared to individuals with ECM/BCM values below median. Linear regression model using ECM/BCM, age, and sex as independent factors, showed ECM/BCM as significantly associated to comorbidity burden (*β* = 0.175, *p* = 0.019); MNA (*β* = −0.285; *p* = 0.004), PNI (*β* = −0.253; *p* = 0.009), GNRI (*β* = −0.363; *p* < 0.0001) and MQI (*β* = −0.311, *p* = 0.0001).

**Discussion:**

Herein, ECM/BCM has been associated, after adjusting for cofounding factors as age and sex, to subjective and objective indexes of health, nutritional and functional status in middle-aged and community dwelling older adults. With an integrated perspective, it could represent an easy to calculate, objective index to assess and monitor health, nutritional and functional status in this population.

## 1 Introduction

Aging is a natural physiological process characterized by a gradual decline in cognitive and physical functions, whose trajectory can be modulated by modifiable factors such as nutrition and dietary habits (1). In older adults, malnutrition is commonly observed and may be a consequence of physiological, psychosocial, socioeconomic factors, existing pathological conditions and polypharmacy (2).

A well-established bidirectional relationship exists between nutritional and health status: poor nutrition can contribute to the onset/worsening of chronic diseases, while a pre-existing clinical condition can affect food intake and energy requirements, further deteriorating nutritional status. This creates a vicious cycle, where malnutrition itself may contribute to disease worsening (3).

Considering all this, assessing the nutritional status in older population is pivotal to prevent disease onset, slow clinical progression, and promote healthy aging (3).

The nutritional status is the result of nutrients intake, absorption, and utilization, and depends on energy homeostasis and organ function. It can be assessed using both subjective and objective measurements (4). The mini nutritional assessment (MNA) questionnaire, validated for older adults, is a widely used and effective tool to screen for malnutrition. It includes not only anthropometric measurements (such as body mass index, calf, and arm circumferences) but also information on involuntary weight loss, appetite and reduced food intake, mobility, neuropsychological disease, polypharmacy, quality of dietary habits, fluids intake and self-perception of nutrition and health status (5). Although it is a valuable tool, its application could be limited in populations with cognitive impairment (6).

Several biochemical markers—including albumin, prealbumin, transferrin and retinol-binding protein—are commonly used to assess protein levels and nutritional status in older adults (7). In this context, the prognostic nutritional index (PNI) and the geriatric nutritional risk index (GNRI) serve as objective and practical tools for assessing nutritional status of patients. Both indices can be calculated using standard laboratory parameters and anthropometric measurements (8, 9). The PNI combines serum albumin concentration and lymphocytes counts, serving as an indicator of both inflammatory and nutritional status (10). Conversely, the GNRI is calculated from serum albumin and body weight, providing a measure of nutritional risk in geriatric population and offering insight into their nutritional status (11).

Overall, clinical tools commonly used to assess nutritional status in older adults are simple and ease to apply but often lack sensitivity in detecting the early-stage malnutrition. For instance, parameters such as body mass index (BMI), serum albumin, and unintentional weight loss can be significantly affected by factors like inflammation, hydration, comorbidity and polypharmacy. As a result, changes such as muscle loss, fat redistribution, or fluid imbalance may go unnoticed (2). These limitations highlight the urgent need for more objective, comprehensive, and integrated approaches to accurately assess malnutrition in the aging population.

Bioimpedance analysis (BIA) is a simple, non-invasive, and accessible method for evaluating changes in body composition commonly associated with aging. A variant of the conventional BIA, the bioelectrical vector impedance analysis (BIVA), enhances the precision of body composition assessment by directly analyzing the two components of the impedance vector (Z): resistance (*R*) and reactance (*Xc*). In BIVA, the position and length of the impedance vector, plotted on the *R-Xc* graph and normalized for height, enable a more accurate evaluation of body cell mass, cellular integrity, and hydration status. This approach offers valuable prognostic information and extends its clinical applicability to populations with altered fluid distribution or hydration status (12).

Among the parameters derived from BIA, phase angle (PhA) has emerged as a clinically relevant marker, reflecting cell mass and membrane integrity. Its validity has been demonstrated across various physiological and pathological conditions (12–14). However, PhA is influenced by factors such as age, sex, and BMI, and may not fully capture the differences in body composition, as distinct impedance vectors can result in similar PhA values. Therefore, while PhA is useful, it may have limitations in detecting subtle changes in hydration when used alone (12, 14, 15).

Under normal physiological condition, approximately 73% of body fluids are distributed within the fat free mass (FFM), which comprises two compartments: body cell mass (BCM) and extracellular mass (ECM). In 1963, Moore and Boyden defined BCM as the cellular components responsible for metabolic “oxygen-requiring, carbon-dioxide-producing” processes (16). BCM represents the total metabolically active, living, functional cellular mass and is a key determinant of basal metabolic rate. It is widely regarded as a crucial indicator of nutritional status (17).

In physiological conditions, ECM and BCM are balanced and expressed as ECM-to-BCM ratio (ECM/BCM) (18). In healthy individuals, ECM/BCM values typically range between 0.85 and 1.00, and deviations from this range may indicate fluid imbalance and/or catabolism (18).

As extensively demonstrated, BCM loss can result from various pathological and/or adaptative mechanisms (14). Quantitative reductions in BCM and cell membrane surface area are observed in conditions such as malnutrition, sarcopenia, and cachexia. Inflammation and oxidative stress-related processes can compromise and damage cell membrane, leading to a functional loss in the number of viable cells (14). An increase in FFM hydration, without change in cell mass, may occur in hemodynamic failure (e.g., heart failure, chronic kidney disease) or in inflammatory states characterized by expansion of extracellular water, the main constituent of ECM (14). In pathological conditions, mechanisms causing both quantitative and qualitative depletion of BCM may coexist with processes that simultaneously promote extracellular fluid accumulation (14).

The ECM/BCM has been primarily studied in pathological conditions (19–22). For instance, in hemodialysis patients, it has been validated as prognostic indicator of mortality risk (22), while in patients with head and neck cancer, it has been proposed as a marker of malnutrition (20).

Despite increasing interest in body fluid distribution parameters such as ECW/ICW, ECW/TBW, and ICW/FFM (23–27), the ECM/BCM remains relatively underexplored in older populations. Derived from estimates of FFM and BCM, it reflects the quality of fat-free mass by quantifying the balance between its functional (BCM) and non-functional (ECM) components, independently of fat mass.

Age-related factors such as oxidative stress, low-grade inflammation, anabolic resistance, which subtend the physiological aging process, may contribute to cellular membrane damage, increased catabolism, and disruption of energy homeostasis. These changes may consequently impair both the quantity and functionality of cells, leading to alterations in body composition and compartment integrity (28).

Therefore, the assessment of ECM/BCM by BIVA may provide valuable insights into age-related conditions such as malnutrition, sarcopenia, systemic inflammation, and fluid overload. Although it relies on sex- and population-specific equations derived from bioimpedance and anthropometric data, ECM/BCM can identify catabolic states and fluid imbalance that may go undetected by other clinical tools, offering a practical and accessible parameter for assessing nutritional and hydration status, especially when interpreted alongside raw bioelectrical data.

In this context, the comprehensive assessment of nutritional status in older adults may benefit from the integration of both traditional and advanced tools, including body composition analysis, biochemical markers, and functional measures to better capture subtle yet clinically relevant changes in physiological reserve and functional decline.

All things considered, the aim of this study was to characterize ECM/BCM in middle-aged adults and community-dwelling older individuals, and to examine its possible associations with indexes of health, nutritional status, and functional performance. To this end, participants underwent standardized clinical, nutritional, and functional assessments using both validated questionnaires and objective measurements.

## 2 Materials and methods

### 2.1 Participants and clinical assessment

A total of 158 adult and older adults (96 women) aged 51–89 years with no medical, physical, or cognitive conditions that could interfere with signing a written consent form or complying with the study protocol, were enrolled in the study. This observational study was approved by the Territorial Ethics Committee (Sardinia Ethics Committee, Prot. PG/2023/5172, 06/04/2023) and conducted in accordance with the Declaration of Helsinki at the Department of Biomedical Sciences, University of Sassari, Italy, from May 2023 to September 2024. Former participants of several studies from the Department of Biomedical Sciences, University of Sassari, were recruited. Public engagement initiatives were also organized to implement recruitment. Written informed consent was obtained from all participants before inclusion and participation to the study. Socio-demographic data were collected through structured interview.

Following enrollment, participants underwent medical history assessment and clinical examination. Participants were excluded if they met any of the following criteria: age < 50 years; diagnosis of malignancy; severe obesity (BMI > 35 kg/m^2^); secondary hypertension; acute cardiovascular or cerebrovascular events within the past 6 months; chronic heart failure or unstable angina; atrial fibrillation; presence of an aortic stent or known abdominal aortic aneurysm; history of organ transplantation; chronic kidney disease, respiratory failure, or liver cirrhosis; moderate-to-severe chronic inflammatory or autoimmune diseases; severe non-compensated metabolic conditions, alcohol or substance dependence; known HIV positivity; or inability to provide informed consent.

Charlson comorbidity index (CCI) was calculated for each participant. The index encompasses 17 comorbid conditions, with diabetes, liver disease, and solid tumors classified into subcategories. Each comorbidity and subcategory is assigned a weight from 1 to 6 accordingly to mortality risk and disease severity. One point is added to the score for each decade of age over 40 years. The total CCI score is obtained by summing of points. A CCI score of 5 is associated with an estimated 10-year survival of 34% (29, 30).

Global cognitive status was assessed using Montreal Cognitive Assessment (MoCA), a widely validated tool used in both clinical practice and research, which comprises 12 subtasks to assess global cognitive function, short-term memory, visuospatial and executive function, attention, language and orientation, concentration and working memory (31). Raw scores were adjusted by adding or subtracting the contribution given by age and education in years. The total MoCA score ranges from 0 to 30, indicating worst or best performance, respectively. Adjusted MoCA scores were considered according to normative values reported by Santangelo and colleagues (32, 33).

### 2.2 Nutritional status assessment

Nutritional status was assessed by trained nutritionists. Anthropometric measurements (such as weight, height, arm and calf circumferences) were collected, and body mass index (BMI, kg/m^2^) was calculated.

The mini nutritional assessment (MNA) questionnaire was administered, and the total score was calculated according to the guidelines (5). The MNA consists of 18 items regarding anthropometry, cognitive and physical disabilities, pharmacotherapy, food and liquids intake, self-perception of nutritional and health status. A total score of 24 or above (up to 30), indicates normal nutrition; a score between 17.0 and 23.5 indicates risk of malnutrition, and a score lower than 17.0 indicates malnutrition (5).

Body composition was assessed using bioelectrical impedance vector analysis (BIVA) with a single-frequency impedance analyzer operating at a frequency of 50 kHz and 250 μA, in accordance with the manufacturer’s instructions (BIA 101 BIVA, Akern, Florence, Italy). Raw bioelectrical parameters were analyzed, and *R*/H and *Xc*/H normalized values were compared with the reference standards for the adult and elderly Italian population (34). Estimated values of FFM, FM, BCM, TBW (as absolute and percentage values) and TBW/FFM were obtained with the data analysis software (BodygramPlus, Akern, Florence, Italy).

ECM/BCM was obtained by subtracting BCM to FFM, both expressed in kg, to obtain ECM in kg. The ratio was calculated by dividing ECM by BCM, both expressed in kg.

### 2.3 Blood parameters

Blood samples were collected at the laboratory of the University Hospital of Sassari for subsequent hematochemical analyses, including, but not limited to, complete blood count, white blood cells (WBC), hematocrit (HTC), hemoglobin (Hgb), total protein, albumin, creatinine and serum iron. PNI and GNRI were calculated as indexes of nutritional and inflammatory (9, 35) and nutritional status (11), respectively.

PNI was obtained using the following formula:


$$\mathrm{PNI}=10^{*}\text{serum albumin}\;(g/\mathrm{dL})+0.005^{*}\text{lymphocyte count}\;(/{\mathrm{mm}}^{3})$$

PNI scores greater than 38 indicate no risk of malnutrition, scores between 35 and 38 indicate moderate risk, and scores below 35 indicate severe risk (35).

GNRI was calculated as follows:


$$\begin{array}{l} \text{GNRI}=(14.89^{*}\text{serum albumin}\;(g/\mathrm{dL}))\\ +(41.7^{*}(\text{current body weight}/\text{ideal body weight})) \end{array}$$

Ideal weight was calculated from Lorentz formula (36):

For men: height (H)-100-[(H-150)/4].

For women: H-100-[(H-150)/2].

For current body weight/ideal body weight >1, the value of 1 was included in the formula (11, 37).

Four grades of nutrition-related risk have been defined. GNRI scores >98 indicate no risk of malnutrition, scores between 92 and 98 indicate low risk, scores between 82 and 92 indicate moderate risk, and scores below 82 indicate severe risk of malnutrition (11).

### 2.4 Muscle strength assessment

Muscle quality index (MQI) is defined as the ratio between muscle strength and muscle mass. Specifically, it has been calculated as best hand grip strength divided by the appendicular skeletal muscle mass (ASMM), both in kg, and used as a variable for the statistical analysis (38, 39).

Hand grip strength was measured using a handgrip dynamometer (G200, Biometrics LTD, Newport, United Kingdom) over three trials, with one-minute rest between trials. The best and average results were recorded. ASMM, the appendicular portion of the skeletal muscle mass, was obtained by BIVA accordingly to Sergi’s et al. (40).

### 2.5 Statistical analysis

The sample size was calculated *a priori* using G*Power software. For an F test employing analysis of covariance (ANCOVA) with fixed effects, main effects, and interactions, a minimum of 128 participants was required to achieve a statistical power (1 − *β* error probability of 0.8), assuming a medium effect size (*f* = 0.25) and a significance level of *α* = 0.05. The corresponding critical *F* value was calculated as 3.9169.

Statistical analyses were performed using SPSS version 20 (SPSS Inc., Chicago, IL, USA). Data are presented as mean ± standard deviation (SD). Data distributions were evaluated graphically using histograms and boxplots for outlier check, by means of descriptive (asymmetry and kurtosis) and statistical methods (Kolmogorov–Smirnov test).

Given the low proportion and random nature of missing data, pairwise deletion was applied for descriptive analyses and one-way analysis of variance (ANOVA), whereas listwise deletion was used for regression and general linear model analyses. Exploratory analyses were conducted to assess the potential confounding role of age, sex, smoking status, and medication use. Variables found to significantly influence ECM/BCM were subsequently included as covariates in the multivariable models to control for their potential confounding effects.

Statistical difference was evaluated by one-way ANOVA with GROUP (sex or age group) as between subject factors. Bonferroni-corrected *post hoc* tests were conducted to test for differences among more than two groups in sociodemographic, nutritional, and biochemical parameters. Participants were classified according to ECM/BCM median of 0.8984 into “low ECM/BCM” group for individuals below the median level and “high ECM/BCM” group for individuals above the median level. General linear model univariate ANOVA was used to evaluate the differences between ECM/BCM groups for the outcomes of interest, including the continuous variable “age” as covariate (ANCOVA). Interaction between ECM/BCM groups*sex was also analyzed.

ECM/BCM was used as continuous variable for the regression analysis. After verifying the absence of multicollinearity (variance inflation factor, VIF < 5, tolerance, and condition index) and influential outliers (studentized residuals, leverage, and Cook’s distance), a linear regression model including sex and age as covariates was conducted to evaluate the association of ECM/BCM with indexes of clinical (CCI), nutritional (MNA, PNI, GNRI), and functional (MQI) status. Differences with a *p* value < 0.05 were considered significant.

## 3 Results

### 3.1 Participants’ sociodemographic, anthropometric and clinical characteristics

A total of 158 subjects (mean age 65.9 ± 7.4 years) were enrolled in the study. The sample included 96 women (60.8%) and 62 men (39.2%). The most represented age decade was “65–75” with 76 subjects (48.1%), followed by “under-65” (≤64 years old) with 64 subjects (40.5%) and “over-75” (≥76 years old) with 18 subjects (11.4%). Sociodemographic, anthropometric, and clinical data are reported in Table 1. Height (F_1,157_ = 196.276; *p* < 0.0001) and weight (F_1,157_ = 75.199; *p* < 0.0001) were significantly different between women and men. Height (F_2,157_ = 5.863; *p* = 0.004) was significantly different among age groups. No significant differences among age groups were observed for weight. Most of the participants (73.1%) had high education level (≥13 years). Twenty-three participants were actual smokers (14.8%).

**Table 1:** Sociodemographic, anthropometric and clinic characteristics of participants.

Demographic	All sample (*n* = 158)	Sex		Age group		
		W (*n* = 96)	M (*n* = 62)	Under 65 (*n* = 64; W, *n* = 37)	65–75 (*n* = 76; W, *n* = 47)	Over 75 (*n* = 18; W, *n* = 12)
Age (y)	65.9 ± 7.4	66.2 ± 7.0	65.4 ± 7.9	58.7 ± 3.6	68.9 ± 2.8	78.7 ± 3.4
Height (cm)	160.2 ± 9.7	154.3 ± 6.0	169.2 ± 7.1^a^	163.1 ± 10.2^b,c^	158.7 ± 8.7	155.8 ± 9.4
Weight (kg)	68.0 ± 12.4	62.4 ± 9.6	76.8 ± 11.0^a^	69.5 ± 14.1	66.8 ± 11.4	68.0 ± 9.3
BMI (kg/m^2^)	26.4 ± 3.5	26.2 ± 3.7	26.7 ± 3.1	25.9 ± 3.7	26.3 ± 3.1	28.1 ± 3.7

Education	All sample (*n* = 152)	W (*n* = 93)	M (*n* = 59)	Under 65 (*n* = 62; W, *n* = 37)	65–75 (*n* = 72; W, *n* = 44)	Over 75 (*n* = 18, W, *n* = 12)
Elementary school	6 (3.9)	5	1	0	2	4
Middle school	35 (23.0)	25	10	14	17	4
High school	70 (46.1)	38	32	29	35	6
Graduate degree	41 (27.0)	25	16	19	18	4

Clinical Index	All sample (*n* = 158)	W (*n* = 96)	M (*n* = 62)	Under 65 (*n* = 64; W, *n* = 37)	65–75 (*n* = 76; W, *n* = 47)	Over 75 (*n* = 18; W, *n* = 12)
CCI	2.9 ± 1.6	3.0 ± 1.7	2.7 ± 1.4	2.0 ± 1.2^b,c^	3.1 ± 1.0^d^	5.1 ± 2.0
MoCA	24.2 ± 3.1	23.8 ± 3.3	24.8 ± 2.8	24.8 ± 2.7^c^	24.1 ± 3.0^d^	21.7 ± 3.8
MNA	27.7 ± 1.8	27.6 ± 1.9	27.9 ± 1.9	27.3 ± 2.0	28.0 ± 1.7	27.8 ± 1.01

Medication	All sample (*n* = 158)	W (*n* = 96)	M (*n* = 62)	Under 65 (*n* = 64; W, *n* = 37)	65–75 (*n* = 76; W, *n* = 47)	Over 75 (*n* = 18; W, *n* = 12)
Antidiabetics	5 (3.2)	2	3	1	2	2
Antihypertensives	47 (29.7)	19	28	9	24	14
Hypolipidemics	30 (19.0)	15	15	6	17	7
Antiplatelets	22 (13.9)	11	11	2	14	6
Anticoagulants	1 (0.6)	0	1	0	1	0

*n*, number; W, women; M, men; y, years; cm, centimeters; kg, kilogram; BMI, Body Mass Index; kg/m^2^, kilogram per square meter; CCI, Charlson Comorbidity Index; MoCA, Montreal Cognitive Assessment. Data are reported as all sample, as age group category and sex. Values are presented as mean ± standard deviation for age, weight, height, BMI, CCI, MoCA. Education (as degree awarded) and medication (as number of subjects, over total subjects, using the reported medications) are expressed as count, n, and percentage (%). Significative difference between women and men^a^. Significative difference among age groups: under 65 and 65–75^b^; under 65 and over 75^c^; 65–75 and over 75^d^.

Mean CCI was 2.9 ± 1.6. CCI was not significantly different between women and men (F_1,157_ = 1.463; *p* = 0.228), but a significant difference was observed among age groups (F_2,157_ = 46.699; *p* < 0.0001). CCI was significantly higher in over 75 (CI: 4.5–5.6) compared to 65–75 (CI: 2.9–3.4) and under 65 individuals (CI: 1.7–2.3) (*p* < 0.0001 and *p* < 0.0001, respectively). Significantly different CCI was observed between 65 and 75 and under 65 individuals (*p* < 0.0001). Drug-treated hypertension (29.7%) and hypercholesterolemia (19.0%) were the most represented chronic diseases. Antiplatelet drugs were used by 13.9% of the sample.

Mean MoCA score was 24.2 ± 3.1. No significant differences between women (CI: 23.1–24.5) and men (CI: 24.0–25.5) were observed (F_1,139_ = 3.299; *p* = 0.072). Significant differences were observed among age groups (F_2,139_ = 6.408; *p* = 0.002). MoCA score was significantly lower in over 75 (CI: 20.2–23.3) compared to 65–75 (CI: 23.4–24.8) and under 65 individuals (CI: 24.1–25.6) (*p* = 0.020 and *p* = 0.001, respectively). No significant differences were observed between 65 and 75 and under 65 individuals.

### 3.2 Nutritional status

MNA score mean was 27.7 ± 1.8 (CI: 27.4–28.0). No significant difference was observed between women (CI: 27.2–28.0) and men (CI: 27.5–28.3) (F_1,155_ = 1.212; *p* = 0.273) and among age groups (F_2,155_ = 2.454; *p* = 0.089).

Hydration mean value, as total body water to fat free mass (TBW/FFM, %), was 73.3 ± 0.3. One-way ANOVA for age group showed significant differences for reactance normalized by height (*Xc*/H), PhA, BCM, ECM/BCM, TBW/FFM. *Post hoc* tests for differences among age groups are reported in Table 2.

**Table 2:** Body composition analysis of participants.

Body composition parameters			Age group			
	All sample		Under 65	65–75	Over 75	
	(*n* = 158; W *n* = 96)	Statistics F_1,157_; *p*	(*n* = 64; W, *n* = 37)	(*n* = 76; W, *n* = 47)	(*n* = 18; W, *n* = 12)	Statistics F_2,157;_ *p*
R/H (Ohm/m)	328.8 ± 60.1	230.985; *p* < 0.0001	321.6 ± 61.3	333.4 ± 61.5	334.9 ± 50.0	0.770; *p* = 0.465
	W 366.0 ± 43.0					
	M 271.1 ± 29.6					
Xc/H (Ohm/m)	33.1 ± 5.1	97.526; *p* < 0.0001	33.5 ± 5.1^a *^	33.5 ± 5.1^b *^	30.1 ± 4.6	3.643; *p* = 0.028
	W 35.7 ± 4.5					
	M 29.2 ± 3.2					
PhA (°)	5.8 ± 0.6	43.378; *p* < 0.0001	6.0 ± 0.6^a #^	5.8 ± 0.6^b #^	5.2 ± 0.5	15.911; *p* < 0.0001
	W 5.6 ± 0.5					
	M 6.2 ± 0.6					
TBW/FFM (%)	73.3 ± 0.3	15.735; *p* < 0.0001	73.3 ± 0.3^a *^	73.3 ± 0.3^b *^	73.5 ± 0.3	3.763; *p* = 0.025
	W 73.2 ± 0.3					
	M 73.4 ± 0.2					
FFM (kg)	49.8 ± 10.1	407.549; *p* < 0.0001	51.7 ± 11.5	48.9 ± 9.4	47.0 ± 6.6	2.100; *p* = 0.126
	W 42.9 ± 4.2					
	M 60.5 ± 6.7					
BCM (kg)	26.4 ± 6.4	351.216; *p* < 0.0001	27.9 ± 7.2^a *^	25.9 ± 5.8	23.1 ± 4.0	4.584; *p* = 0.012
	W 22.1 ± 2.7					
	M 33.0 ± 4.6					
ECM (kg)	23.4 ± 4.1	283.427; *p* < 0.0001	23.8 ± 4.5	23.0 ± 3.9	23.9 ± 3.1	0.665; *p* = 0.516
	W 20.8 ± 2.1					
	M 27.5 ± 2.8					
ECM/BCM ratio	0.91 ± 0.12	36.516; *p* < 0.0001	0.87 ± 0.10^a #^	0.91 ± 0.11^b #^	1.04 ± 0.12	18.263; *p* < 0.0001
	W 0.95 ± 0.11					
	M 0.84 ± 0.11					

*n*, number; W, women; M, men; R, resistance; Xc, reactance; H, height; PhA, phase angle; TBW, total body water; FFM, fat free mass; BCM, body cell mass; ECM, extracellular mass. Data are reported as all sample, sex and age group category. Values are presented as mean ± standard deviation. Sex and age-group differences in all sample were evaluated by ONE-WAY ANOVA and post-hoc Bonferroni for age-group differences. ^#^For values of *p* < 0.0005; *For values of *p* < 0.05. Significative difference among age groups: under 65 and over 75^a^; 65–75 and over 75^b^.

Overall, body composition parameters were significantly different between men and women, as reported in Table 2.

### 3.3 Blood parameters

Sex differences were found for all the parameters except for total protein and albumin, with men presenting significantly higher values than women (Table 3). Significant differences were observed among age groups with WBC higher in over 75 (CI: 5.9–7.5) compared to individuals aged 65–75 (CI: 5.4–5.9) (*p* = 0.013); hematocrit lower in over 75 (CI: 39.5–43.2) compared to individuals aged 65–75 (CI: 43.1–44.8) (*p* = 0.023); albumin lower in over 75 (CI: 4.0–4.2) compared to under 65 (CI: 4.2–4.3) (*p* = 0.009) and individuals aged 65–75 (CI: 4.2–4.3) (*p* = 0.014); serum iron lower in over 75 (CI: 54.7–87.1) compared to under 65 individuals (CI: 88.3–102.3) (*p* = 0.006) and individuals aged 65–75 (CI: 85.4–97.4) (*p* = 0.025). PNI and GNRI were not significantly different between women and men. Only PNI showed significant differences among age groups with lower index in over 75 (CI: 39.7–42.1) compared to individuals aged 65–75 (CI: 42.2–43.2) (*p* = 0.014) and under 65 individuals (CI: 42.3–43.3) (*p* = 0.009) (Table 3).

**Table 3:** Clinical blood parameters of participants.

Clinical blood parameters			Age group			
	All sample		Under 65	65–75	Over 75	
	(*n* = 154; W *n* = 95)	Statistics	(*n* = 62; W *n* = 36)	(*n* = 76; W *n* = 47)	(*n* = 16; W *n* = 11)	Statistics
WBC (*n*°/μl)	5.9 ± 1.4	F_1,153_ = 8.073; *p* = 0.005	5.9 ± 1.5	5.7 ± 1.1^b*^	6.7 ± 1.5	F_2,153_ = 4.269; *p* = 0.016
	W 5.6 ± 1.2					
	M 6.3 ± 1.5					
Hemoglobin (g/dL)	13.9 ± 1.3	F_1,153_ = 40.538; *p* < 0.0001	14.0 ± 1.1^a*^	14.0 ± 1.4^b*^	13.1 ± 1.3	F_2,153_ = 3.781; *p* = 0.025
	W 13.4 ± 1.1					
	M 14.7 ± 1.3					
Hematocrit (%)	43.6 ± 3.6	F_1,153_ = 52.964; *p* < 0.0001	43.7 ± 3.1	44.0 ± 3.8^b^	41.4 ± 3.5	F_2,153_ = 3.697; *p* = 0.027
	W 42.2 ± 3.1					
	M 45.9 ± 3.1					
Total protein (g/dL)	7.1 ± 0.4	F_1,152_ = 0.274; *p* = 0.601	7.1 ± 0.4	7.1 ± 0.4	7.0 ± 0.4	F_2,152_ = 1.103; *p* = 0.335
	W 7.1 ± 0.4					
	M 7.1 ± 0.4					
Albumin (g/dL)	4.3 ± 0.2	F_1,152_ = 1.377; *p* = 0.242	4.3 ± 0.2^a*^	4.3 ± 0.2^b*^	4.1 ± 0.2	F_2,152_ = 4.770; *p* = 0.010
	W 4.2 ± 0.2					
	M 4.3 ± 0.2					
Serum Iron (μg/dL)	90.9 ± 27.7	F_1,151_ = 7.453; *p* = 0.007	95.3 ± 27.7^a *^	91.4 ± 26.1^b *^	70.9 ± 29.2	F_2,151_ = 4.918; *p* = 0.009
	W 86.2 ± 24.4					
	M 98.6 ± 31.1					
PNI	42.5 ± 2.2	F_1,152_ = 1.377; *p* = 0.243	42.8 ± 2.0^a*^	42.7 ± 2.2^b*^	40.9 ± 2.2	F_2,152_ = 4.775; *p* = 0.010
	W 42.4 ± 2.0					
	M 42.8 ± 2.4					
GNRI	104.7 ± 3.5	F_1,152_ = 1.948; *p* = 0.165	105.0 ± 3.4	104.9 ± 3.6	102.6 ± 3.2	F_2,152_ = 3.011; *p* = 0.052
	W 104.4 ± 3.4					
	M 105.2 ± 3.7					

*n*, number; W, women; M, men; WBC, white blood cell; PNI, prognostic nutritional index (10 × albumin (g/dL)) + (0.005 × total lymphocytes value (μl)); GNRI, geriatric nutritional risk index (14.89 × serum albumin (g/dL)) + (41.7 × (current body weight/ideal body weight)). Data are reported as all sample, sex and age group category. Values are presented as mean ± standard deviation. Sex and age-group differences in all sample were evaluated by ONE-WAY ANOVA and *post-hoc* Bonferroni for age-group differences. *For values of *p* < 0.05. Significative difference among age groups: under 65 and over 75^a^; 65–75 and over 75^b^.

### 3.4 Muscle strength assessment

MQI mean value was 1.4 ± 0.3. A significant difference was observed between women (1.3 ± 0.2; CI: 1.3–1.4) and men (1.6 ± 0.3; CI: 1.5–1.7) (F_1,157_ = 48.653; *p* < 0.0001). Significant differences were also observed among age groups (F_2,157_ = 7.987; *p* < 0.0001). *Post hoc* tests detected a significantly lower MQI in over 75 (1.2 ± 0.2; CI. 1.1–1.3) compared to 65–75 (1.4 ± 0.3; CI: 1.4–1.5) (*p* = 0.001) and under 65 individuals (1.4 ± 0.2; CI: 1.4–1.5) (*p* = 0.001). No significant differences were observed between 65–75 and under 65 individuals.

### 3.5 Extracellular mass to body cell mass ratio analysis

#### 3.5.1 ECM/BCM characterization

ECM/BCM mean value was 0.91 ± 0.12. ECM/BCM was found higher in women (CI: 0.93–0.97) compared to men (CI: 0.82–0.87). ECM/BCM was significantly different among age groups, and Bonferroni *post hoc* test showed significantly higher ECM/BCM in individuals aged over 75 (CI: 0.99–1.10) compared to 65–75 (CI: 0.88–0.93) (*p* < 0.0001), and under 65 (CI: 0.84–0.90) (*p* < 0.0001) (Table 2). No significant differences in ECM/BCM were observed between smokers and non-smokers, nor in relation to the use of antihypertensive, hypolipidemic, antidiabetic, or antiplatelet medications (all *p* > 0.05). For this reason, these factors were not included in the multivariable models to adjust for their potential influence.

Participants were classified by ECM/BCM median in 2 groups: “low ECM/BCM” (79 subjects, 31 women) and “high ECM/BCM” (79 subjects, 65 women) (Table 4). Estimated marginal means of the outcomes of interest, corrected by the covariate age (mean 65.97), were significantly different between the two ECM/BCM groups, as shown in Table 5. Comorbidity index was not significantly different between the two groups after covariate correction. ECM/BCM group*sex interaction was observed only for the outcome MQI (F_1,157_ *=* 4.729; *p* = 0.031).

**Table 4:** ECM/BCM groups characterization accordingly to sex and age groups.

ECM/BCM group				Age group		
		All sample	Sex	Under 65	65–75	Over 75
		*n* = 158	W, *n* = 96	(*n* = 64; W, *n* = 37)	(*n* = 76; W, *n* = 47)	(*n* = 18; W, *n* = 12)
Low ECM/BCM	Frequence	79	W, *n* = 31	39 (W, *n* = 15)	38 (W, *n* = 15)	2 (W, *n* = 1)
	ECM/BCM	0.81 ± 0.06	W 0.84 ± 0.04	0.80 ± 0.06	0.82 ± 0.05	0.82 ± 0.07
			M 0.80 ± 0.06			
High ECM/BCM	Frequence	79	W, *n* = 65	25 (W, *n* = 22)	38 (W, *n* = 32)	16 (W, *n* = 11)
	ECM/BCM	1.00 ± 0.09	W 1.00 ± 0.09	0.97 ± 0.05	0.99 ± 0.09	1.07 ± 0.09
			M 1.00 ± 0.10			

*n*, number; W, women; M, men; ECM/BCM, extracellular mass to body cell mass ratio. High ECM/BCM and low ECM/BCM groups include individuals above and below median (0.8984), respectively. ECM/BCM ratio values are presented as mean ± standard deviation. Frequence of ECM/BCM group in all sample, in relation to sex and to age groups is expressed as count, *n*.

**Table 5:** Differential analysis for clinical, nutritional and functional indexes accordingly to ECM/BCM group and sex.

Indexes	All sample	ECM/BCM group		Statistics	
		Low ECM/BCM	High ECM/BCM	ECM/BCM group main effect	ECM/BCM group*sex interaction
CCI	2.847 (2.628–3.067)	2.792 (2.517–3.067)	2.903 (2.547–3.258)	F_1,157_ = 0.227; *p* = 0.634	F_1,157_ = 0.113; *p* = 0.738
MNA	27.803 (27.479–28.127)	28.147 (27.740–28.553)	27.460 (26.935–27.984)	F_1,155_ = 3.991; ***p*** **= 0.048**	F_1,155_ = 1.591; *p* = 0.209
PNI	42.401 (42.005–42.796)	43.052 (42.567–43.538)	41.749 (41.106–42.392)	F_1,152_ = 9.847; ***p* = 0.002**	F_1,152_ = 1.757; *p* = 0.187
GNRI	104.532 (103.9–105.2)	105.660 (104.877–106.443)	103.403 (102.366–104.441)	F_1,152_ = 11.355; ***p* = 0.001**	F_1,152_ = 1.349; *p* = 0.247
MQI	1.420 (1.378–1.461)	1.493 (1.441–1.545)	1.347 (1.279–1.414)	F_1,157_ = 11.081; ***p* = 0.001**	F_1,157_ = 4.729; ***p* = 0.031**

ECM/BCM, extracellular mass to body cell mass ratio. High ECM/BCM and low ECM/BCM groups include individuals above and below median (0.8984), respectively. CCI, Charlson Comorbidity Index; MNA, mini nutritional assessment; PNI, prognostic nutritional index; GNRI, geriatric nutritional risk index; MQI, muscle quality index. Estimated marginal mean (95% CI), corrected by the covariate “age,” are reported for all sample and ECM/BCM group category. General linear model ANCOVA was performed to evaluate ECM/BCM group main effect and ECM/BCM group*sex interaction. Bold values for *p* < 0.05.

#### 3.5.2 Linear regression analysis

Linear regression was performed to evaluate the prediction of dependent variables as CCI, MNA, PNI, GNRI, and MQI using ECM/BCM and age and sex as confounder factors. No multicollinearity was observed (all VIF < 2) and no influential outliers (accordingly to studentized residuals and Cook’s distances) were revealed. Overall, the regression models were statistically significant for all the examined indices.

The model for CCI was highly significant (F_3,157_ = 42.837, *p* < 0.0001) and explained 45.5% of the variance (*R*^2^ = 0.455; *R*^2^_adj_ = 0.444). The model for MNA was also significant (F_3,155_ = 4.155, *p* = 0.007), explaining the 7.6% of the variance (*R*^2^ = 0.076; *R*^2^_adj_ = 0.058). For the PNI, the model reached statistical significance (F_3,152_ = 5.195, *p* = 0.002), with 9.5% of the variance explained (*R*^2^ = 0.095; *R*^2^_adj_ = 0.076). Similarly, GNRI was significant (F_3,152_ = 7.417, *p* < 0.0001), accounting for 13.0% of the variance (*R*^2^ = 0.130; *R*^2^_adj_ = 0.112). Finally, the regression model for MQI demonstrated strong significance (F_3,157_ = 25.387, *p* < 0.0001), explaining 33.1% of the variance (*R*^2^ = 0.331; *R*^2^_adj_ = 0.318). ECM/BCM was shown a significant predictor for all investigated outcomes, as shown in Table 6.

**Table 6:** Linear regression analysis for the prediction of dependent variables CCI, MNA, PNI, GNRI and MQI by predictors ECM/BCM, sex and age.

Dependent variable	Model	*B*	SE	*β*	*t*	*p*	VIF
CCI	ECM/BCM	2.273	0.962	0.175	2.364	0.019	1.548
	Age	0.123	0.014	0.582	8.726	0.0001	1.259
	Sex	0.038	0.213	0.012	0.180	0.857	1.267
MNA	ECM/BCM	−4.238	1.441	−0.285	−2.940	0.004	1.545
	Age	0.059	0.021	0.241	2.753	0.007	1.263
	Sex	−0.075	0.320	−0.021	−0.235	0.814	1.259
PNI	ECM/BCM	−4.637	1.746	−0.253	−2.655	0.009	1.491
	Age	−0.033	0.026	−0.111	−1.284	0.201	1.219
	Sex	−0.076	0.390	−0.017	−0.195	0.846	1.259
GNRI	ECM/BCM	−10.745	2.759	−0.363	−3.895	0.0001	1.491
	Age	−0.015	0.041	−0.030	−0.361	0.718	1.219
	Sex	−0.316	0.617	−0.044	−0.512	0.609	1.259
MQI	ECM/BCM	−0.697	0.184	−0.311	−3.789	0.0001	1.548
	Age	−0.002	0.003	−0.049	−0.669	0.504	1.259
	Sex	0.192	0.041	0.349	4.710	0.0001	1.267

ECM/BCM, extracellular mass to body cell mass ratio; CCI, Charlson Comorbidity Index; MNA, mini nutritional assessment; PNI, prognostic nutritional index; GNRI, geriatric nutritional risk index; MQI, muscle quality index. VIF, variance inflation factor. Statistical analysis is reported as non-standardized beta (*B*), standard error (SE), standardized beta (*β*), *t*-test (*t*) and significance value (*p*).

## 4 Discussion

This study investigated the association between ECM/BCM, an index obtained by BIVA, and both subjective and objective indicators of nutritional, health and functional status, in middle-aged adults and community dwelling older individuals. The findings demonstrated that ECM/BCM increases with age and is higher in women than in men, and that high ECM/BCM values are associated with lower scores of MNA, PNI, GNRI, and MQI. Furthermore, regression analyses revealed that ECM/BCM was a significant predictor of health, nutritional, and functional status. This association remained statistically significant even after adjusting for potential confounders such as age and sex.

Herein, ECM/BCM is proposed as an indicator of FFM quality in older individuals, reflecting cellular and extracellular mass balance. ECM/BCM mean values were found to fall within the normality range, according to Talluri et al. (18). Single values below (31.6%) and above this range (20.9%) were principally observed in men and women, respectively. As expected, the ratio varied accordingly to sociodemographic characteristics such as age and sex, with values increasing with age and being higher in women than in men. These findings are consistent with those of Gallagher et al. who reported an age-related decline of BCM relative to FFM. Notably, this decline was less pronounced in women than in men, suggesting that sex may moderate the effect of aging on body composition. Furthermore, the study further suggests that the reduction of BCM relative to FFM with advancing age is accompanied by a small but consistent increase in extracellular fluid (41, 42).

After controlling for age and sex, ECM/BCM proves to be a predictor of comorbidity burden, by means of CCI, even when not major health problems are present, suggesting its potential utility in detecting early sign of clinical distress. This finding is consistent with a previous prospective observational study by Ruperto and Barril, who reported a positive correlation between ECM/BCM and CCI in hemodialyzed patients. In the same study, an ECM/BCM cut-off value ≥1.20, in conjunction with elevated inflammatory markers and comorbidities, was significantly associated with an increased risk of all-cause mortality (22).

Although the majority of the participants in this study did not exhibit major clinical conditions, except for pharmacologically treated hypertension (29.7%) and hypercholesterolemia (19.0%), and demonstrated hydration status (TBW/FFM %) within the normality range (18, 34), age-related processes, such as protein catabolism, chronic inflammation, oxidative stress, and mitochondrial dysfunction, may subtend the observed association between ECM/BCM and the clinical phenotype (43).

Interestingly, several studies have explored the relationship between reduction in whole body lean mass and cognitive decline (6, 13). A recent study involving segmental bioimpedance analysis in 365 cognitively normal individuals, 123 patients with mild cognitive impairment, and 30 patients with Alzheimer Disease, demonstrated a negative correlation between extracellular-to-intracellular water ratio, in the lower extremities, and cognitive impairment associated to Alzheimer’s disease (44).

In this study, participants exhibit generally preserved cognitive functions, although significative lower MoCA score in over 75 compared to younger individuals were found (32, 33). This relative preservation of cognitive status likely limited the analysis of an association between ECM/BCM and cognitive performance. Further studies are warranted to investigate this potential relationship in population with cognitive impairment.

Analysis of MNA scores indicate an overall good nutritional status among participants, with no differences across age groups or between sexes. Individuals with higher ECM/BCM values presented lower MNA scores, although these scores remained within the normal nutritional status range (5). This trend was reflected by the modest yet statistically significant association between ECM/BCM and nutritional status as assessed by MNA.

Our findings are consistent with previous studies reporting a positive association between MNA and PhA (6) a parameter reflecting cell membrane integrity, body cell mass, which is closely related to body fluid distribution, and compartmentalization (37, 45). However, PhA represents a composite single value that is significantly influenced by age, sex, and BMI and does not provide specific insights into shifts between BCM and ECM, thus requiring a combined analysis of body compartments estimates (14). Given its strong correlation with PhA and its predictive value for MNA score, as demonstrated by our linear regression model, ECM/BCM may serve as a simpler yet informative BIVA-derived parameter for assessing nutritional status in older adults.

Biochemical data presented in this study further support the relationship between ECM/BCM and nutritional status. GNRI and PNI are widely used indicators of nutritional status and have been associated to diverse clinical outcomes (8–10, 37, 46). Herein, individuals with higher ECM/BCM exhibited significantly lower PNI and GNRI values compared to those with lower ECM/BCM, even after adjusting for age and sex. The association were confirmed by the regression model, which identified ECM/BCM as a significant predictor of PNI and GNRI.

Although blood parameters and nutritional index scores were within normal ranges in our study population (11, 35, 47, 48), the association between higher ECM/BCM and lower PNI and GNRI may reflect an early loss of BCM and fluid expansion in older individuals, potentially linked to lower level of albumin. Albumin is the most used plasma biomarker for assessing malnutrition (49) and hypoalbuminemia has been associated to low muscle mass and function, increased mortality risk, and poor recovery outcomes both in community-dwelling and hospitalized older adults (47). Our findings are confirmed by a previous work by Rondanelli and co-authors who reported a significant association between the body cell mass index (BCM/(height)^2^) and serum albumin, in a geriatric population (50). In line with our results, Ruperto and Barril reported a significant correlation between ECM/BCM and both malnutrition–inflammation score and high sensitivity C-reactive protein (s-CRP) in hemodialyzed patients. A more recent study investigating the relationship between body composition and inflammatory markers, found an inverse association between BCM and absolute FFM with s-CRP (51). The authors explained this correlation by the activation of myokines in skeletal muscle to induce protectory anti-inflammatory activity (51).

MQI has been proposed as a better predictor of functional capacity in older individuals compared to muscle mass alone or hand-grip strength (52). In hemodialyzed patients, MQI has also been associated with clinical prognosis, and it has been shown that visceral fat does not significantly influence this relationship (53). These findings underscore the relevance of FFM and its two components, in relation to functional capacity as expressed by MQI.

The data presented in this study identify ECM/BCM as a significant predictor of MQI in older adults. Notably, high ECM/BCM were associated with lower MQI, even after adjusting for potential confounding factors, and a significant interaction with sex was observed. Specifically, sex-related differences in MQI were evident across both ECM/BCM groups but were more pronounced in the low ECM/BCM group. This finding may be attributed to baseline sex differences in the proportion of BCM relative to FFM, which tend to diminish as age increases, with men experiencing a more accelerated decline in BCM compared to women (41).

Several studies support our findings. Specifically, the association between FFM and its components, in terms of BCM/FFM, ECW/ICW, ECW/TBW, with physical performance in both adult and older individuals has been previously demonstrated. In community dwelling elderly women, hand-grip strength and gait speed was found to be negatively correlated with the total body ECW/ICW ratio, which has been suggested as an indicator of overall health status in this population (54). Serra-Prat and co-authors suggested that the decline in ICW observed in older adults, potentially reflecting both a reduction in muscle cell number and decrease cellular hydration, is associated with reduced muscle strength and impaired functional performance (55). Similarly, Yamada et al. demonstrated in a cohort of 115 adults that both BCM/FFM and ECW/ICW were significant predictors of peak oxygen uptake, a key indicator of cardiorespiratory fitness (24).

From a mechanistic perspective, the association between ECM/BCM and health and functional status in older adult, may be driven by age-related malnutrition. This condition results from physiological changes that affect dietary requirement, nutrients absorption and metabolic utilization, accompanied by imbalances in energy, protein and micronutrients intake, ultimately leading to catabolism and altered body composition (56).

In this context, the interplay between nutrition and immune function may represent a critical factor particularly in older individuals. At a cellular level, immune-senescence and inflammation are the key processes associated with impaired immune responses and a pro-inflammatory milieu, which underlie many of the physiological and pathological changes observed with aging (57).

This study presents some limitations. The cross-sectional design prevents from clarifying the causal association between ECM/BCM and health, nutritional, and functional status. The absence of data related to nutritional/energy intake and level of physical activity did not allow to verify the presence of poor dietary habits and sedentary behavior in our population. Future studies, specifically designed, should further investigate the relationship between ECM/BCM and lifestyle, psychosocial, and environmental factors. The absent of overt malnutrition cases within the sample limited the ability to define a cut-off point for discriminating nutritional and functional status. Moreover, the relatively small sample size and single-center design may limit the generalizability of the findings to other populations. Future multi-centered, cross-cultural studies are warranted to validate ECM/BCM and to further evaluate its universality and reliability in the diverse population groups. While pairwise and listwise deletion were applied, due to the low proportion of missing values and the nature of the available data, the use of multiple imputation could be considered in future studies to further reduce the risk of bias and enhance statistical efficiency. BIA is not considered the gold standard for body composition measurements because its reliance on population specific equations to calculate body composition estimates, might lead to errors in older and diseased subjects, who are characterized by altered body composition and hydration status. The application of BIVA overcomes this limitation, since it is independent of predictive equations and necessary assumptions to estimate body compartment through the analysis of raw bioelectrical parameters, it provides semi-quantitative data on body cell mass, cellular integrity and tissue hydration status (12). ECM/BCM is derived from estimates of FFM and BCM, calculated using age- and sex-specific equations based on bioelectrical impedance and anthropometric data. Despite this, it remains a practical and accessible indicator of nutritional and hydration status, particularly when interpreted alongside raw bioelectrical data. Unlike vector analysis, which requires expertise, ECM/BCM also may represent a relatively simple and accessible parameter, both for clinician to interpret and for patients to understand. Furthermore, compared to dual-energy X-ray absorptiometry, the gold standard for body composition assessment, BIVA offers an easy to use, portable, not expensive and non-invasive alternative for the assessment and monitoring of body composition (12).

In conclusion, clinical guidelines recommend the use of standardized screening tools to identify the risk of malnutrition or the presence of overt malnutrition in older individuals, along with the collection of objective measurements, including anthropometric, biochemical, nutritional and functional markers (58). These recommendations underlying the need of adopting multidimensional approaches to better characterize aging phenotypes and risk factors.

In this study, ECM/BCM was found to be associated, after adjustment for cofounding factors as age and sex, with both subjective and objective indicators of health, nutritional and functional status in middle-aged adults and community dwelling older individuals. From an integrated perspective, ECM/BCM may serve as a simple, objective, and easily obtainable index for the assessment and monitoring of health, nutritional and functional status in this population.

From this perspective, an ECM/BCM imbalance may reflect compromised health, nutritional, and functional status in older individuals, who are intrinsically frail, even in the absence of overt clinical conditions, as observed in our sample. This imbalance may represent the physiological progressive loss of homeostatic resilience in response to stress. Future research will be addressed to characterize ECM/BCM shifts in both physiological aging and age-related clinical conditions and to elucidate the underlying pathophysiological mechanisms (e.g., low-grade chronic inflammation, oxidative stress, etc.) that may contribute to ECM/BCM imbalance and its association with functional decline in both aging and age-related diseases.

## Data Availability

The data that support the findings of this study are made available from the corresponding author, upon reasonable request.

## Glossary

ASMM: appendicular skeletal muscle mass
BCM: body cell mass
BIVA: bioelectrical impedance vector analysis
BMI: body mass index
CCI: Charlson comorbidity index
ECM: extracellular mass
ECM/BCM: extracellular mass to body cell mass ratio
ECW: extracellular water
ECW/ICW: extracellular-water–intracellular-water ratio
FFM: fat free mass
FM: fat mass
GNRI: geriatric nutritional risk index
Hgb: hemoglobin
HTC: hematocrit
ICW: intracellular water
MNA: mini nutritional assessment
MoCA: Montreal cognitive assessment
MQI: muscle quality index
PhA: phase angle
PNI: prognostic nutritional index
R/H: resistance standardized by height
TBW: total body water
TBW/FFM: total body water/fat-free mass ratio
WBC: white blood cells
Xc/H: reactance standardized by height
